# Art reaches within: aesthetic experience, the self and the default mode network

**DOI:** 10.3389/fnins.2013.00258

**Published:** 2013-12-30

**Authors:** Edward A. Vessel, G. Gabrielle Starr, Nava Rubin

**Affiliations:** ^1^Center for Brain Imaging, New York UniversityNew York, NY, USA; ^2^Department of English, New York UniversityNew York, NY, USA; ^3^Center for Neural Science, New York UniversityNew York, NY, USA; ^4^ICREA and DTIC, Universitat Pompeu FabraBarcelona, Spain

**Keywords:** neuroaesthetics, art, default mode network, self-relevance, medial prefrontal cortex (mPFC), fMRI, visual, individual differences

## Abstract

In a task of rating images of artworks in an fMRI scanner, regions in the medial prefrontal cortex that are known to be part of the default mode network (DMN) were positively activated on the highest-rated trials. This is surprising given the DMN's original characterization as the set of brain regions that show greater fMRI activity during rest periods than during performance of tasks requiring focus on external stimuli. But further research showed that DMN regions could be positively activated also in structured tasks, if those tasks involved self-referential thought or self-relevant information. How may our findings be understood in this context? Although our task had no explicit self-referential aspect and the stimuli had no *a priori* self-relevance to the observers, the experimental design we employed emphasized the personal aspects of aesthetic experience. Observers were told that we were interested in their individual tastes, and asked to base their ratings on how much each artwork “moved” them. Moreover, we used little-known artworks that covered a wide range of styles, which led to high individual variability: each artwork was rated highly by some observers and poorly by others. This means that rating-specific neural responses cannot be attributed to the features of any particular artworks, but rather to the aesthetic experience itself. The DMN activity therefore suggests that certain artworks, albeit unfamiliar, may be so well-matched to an individual's unique makeup that they obtain access to the neural substrates concerned with the self—access which other external stimuli normally do not get. This mediates a sense of being “moved,” or “touched from within.” This account is consistent with the modern notion that individuals' taste in art is linked with their sense of identity, and suggests that DMN activity may serve to signal “self-relevance” in a broader sense than has been thought so far.

## Introduction

The burgeoning field of **neuroaesthetics** attempts to address the mysteries of the human preoccupation with art by studying the underlying brain mechanisms. And, while understanding the artistic creative process itself is certainly a formidable challenge, many of the open questions concern the *response* to works of art by their viewers, listeners, and readers. What makes us so drawn to certain artistic creations, so influenced and moved by them? In recent years, we have learned a considerable amount from brain imaging studies about the neural correlates of **aesthetic experience** and how they relate to sensory, reward, and emotion neural processes (for reviews see Di Dio and Gallese, 2009; Brown et al., 2011; Chatterjee, 2011; Nadal and Pearce, 2011). One aspect that has so far received little investigation is that of individual differences: although it is widely recognized that individuals can differ markedly in their aesthetic response, previous research in neuroaesthetics tended to utilize art pieces that were manipulated in a manner intended to have a consistent effect on observers' preferences or that were generally highly regarded and often, widely known (e.g., the *Mona Lisa*). It seems reasonable to expect that studying widely admired artwork can help uncover the universal aspects of aesthetic experience. But studying artworks that generate a diversity of responses can also be valuable. Brain imaging can, in principle, be used to probe the neural correlates of an experience in a manner dissociable from the external stimuli that gave rise to this experience. In particular, it is possible to *capitalize* on the differences in individual's responses to artworks to search for commonalities in brain activity associated with the aesthetic experience itself, irrespective of the stimulus properties of specific works of art that gave rise to it. We have used this strategy in a recent study (Vessel et al., 2012) and the results underscore its power and promise, by confirming known results while at the same time revealing new and hitherto unsuspected findings.

KEY CONCEPT 1 | Neuroaesthetics A multi-disciplinary field aimed at understanding the neural basis of aesthetic experience and behavior. This includes interactions with art-objects as well as aesthetic modes of interaction with non-art objects, such as faces, natural objects, and scenes.

KEY CONCEPT 2 | Aesthetic experience Aesthetics is a discipline concerned with the perception, appreciation, and production of art. Aesthetic experiences, such as looking at paintings, listening to music or reading poems, are linked to the perception of external objects, but not to any apparent functional use the objects might have. Aesthetic experience involves more than preference, encompassing a variety of emotional responses ranging from beauty to awe, sublimity, and a variety of other (often knowledge-based) emotions.

## Highly individualized responses to visual art

As in much previous work in neuroaesthetics, we wished to compare fMRI brain activity during observation of visual art that elicited a high level of aesthetic appreciation with responses to unappreciated artworks. But there was an important difference: a primary goal of our study was to move away from the scenario whereby different observers tend to respond similarly to the art presented to them. (The rationale for this goal is explained below, section *Neural Correlates of Aesthetic Appreciation: Two Distinct Activity Patterns*). To achieve this, we collated a set of images of two-dimensional visual artwork spanning a wide variety of periods, regions, styles and genres (fifteenth to twentieth century, Western and Eastern works, including a range of representational and abstract genres). Importantly, although the images were taken from museum collections, the artworks were not commonly reproduced and were therefore novel to our observers. Moreover, the instructions to the participants emphasized that we were interested in their own, individual response (rather than in what may be the “normative” assessment of each artwork), and that aesthetic experiences may come in a variety of forms: “The paintings may cover the entire range from ‘beautiful’ to ‘strange’ or even ‘ugly.’ Respond on the basis of how much this image *moves* you.” Each observer (*N* = 16) was shown the same series of 109 color artworks (in randomized order) while being scanned using fMRI, and was asked to rate each artwork on a 4-point scale according to these instructions. For a list of artworks and other experimental details, see Vessel et al. (2012), *Materials and Methods* and *List of Artworks*.

**Figure 1:**
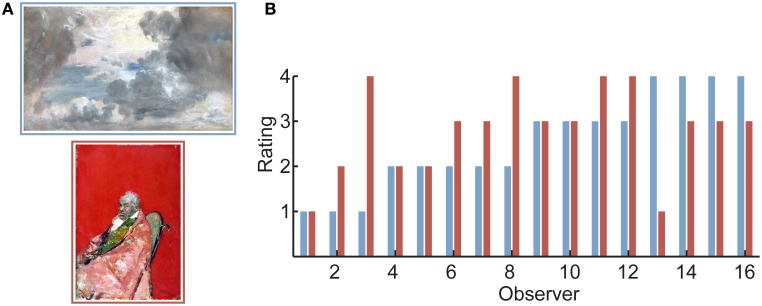
**Aesthetic appreciation of visual art is highly individual. (A)** Two sample images from the set observers were shown. Images were reproductions of museum artworks that are not commonly reproduced (see *Acknowledgments* for image credits). Observers rated each image for how much the artwork “moved” them on a scale of 1 (lowest) to 4 (highest). **(B)** Ratings of all 16 observers for the two images in **(A)**. As was typical for the artworks used in the experiment, observers differed widely in their response to the pair of images. In particular, some observers rated the top image (blue bars) to be highly moving, while others rated the bottom image (red bars) to be highly moving. (For this bar plot, observers were first sorted by their rating to the top image, then by their rating to the bottom image).

Analysis of the behavioral responses revealed that responses were indeed highly individual: there was little agreement between observers regarding how moving each painting was (0.13 average correlation between the ratings of pairs of observers, computed over the entire set of images; *SD* = 0.17). This means that, on average, each image was rated as highly moving by one subset of observers and rated poorly by another subset of observers (Figure 1). These results stand in contrast with the rather high agreement obtained when observers make preference judgments for real-world scenes [e.g., 0.46 between-observer correlation in Vessel and Rubin (2010)] or attractiveness judgments for faces [0.41 correlation between pairs of strangers in Bronstad and Russell (2007); 0.40 in Honeköpp (2006)]. As we shall see below, the low agreement between individuals in terms of their aesthetic response is what allowed us to disentangle the external attributes of specific stimuli from the internal (neural) states to which they gave rise.

Another finding from the behavioral data that will play a role in interpreting the brain imaging results is that, on average, observers used the highest (“4”) rating significantly less than 25% of the time (mean: 16.7%; *SD* = 11.6%; 4 of 16 observers gave more than 25% “4” responses). This is interesting given that there was no special mention of the highest rating in our instructions, and that in rating sensory/perceptual attributes (e.g., perceived brightness) observers tend to distribute their responses across all available options. That the observers in our experiments behaved differently, and did not calibrate their responses so as to give a rating of “4” to roughly a quarter of the stimuli, suggests that they reserved this response for images which met a certain internal (and generally high) criterion.

## Neural correlates of aesthetic appreciation: two distinct activity patterns

The fMRI data were analyzed to compare responses during trials in which the artworks were highly-rated with trials of low-rated artwork. Contrasting brain activity between conditions that differ by the observers' own responses, or performance, has been used successfully in many domains of cognitive neuroscience (e.g., studying neural correlates of memory encoding by contrasting activity in subsequently-remembered and forgotten trials; Brewer et al., 1998; Wagner et al., 1998). But in the context of neuroaesthetics, extra care must be taken to dissociate neural correlates of the aesthetic experience itself from other aspects of brain activity elicited by the stimuli. As a simple example, suppose observers are presented with a set of paintings comprised mainly of portraits and landscapes, and suppose further that most of them happen to appreciate portraiture more than landscapes. Face-selective brain regions would then likely show up in a contrast between highly-rated and low-rated trials, but is it warranted to interpret their activity as pertaining to aesthetic experience? In this case, the (conjured) agreement in aesthetic preference is simple enough, and our knowledge of face-selectivity in the brain sound enough, to easily discern that the activity can be explained by other aspects of the stimuli (the types of objects depicted). But in fact, such potential confounds are present whenever there is high agreement between observers about the art: the highly-rated and low-rated trials in such cases correspond to different sets of (artwork) stimuli, which may well result in some differential activation unrelated to the aesthetic experience they produce. Conversely, high variability between different observers' aesthetic judgments alleviates the potential confound: in the limit of completely uncorrelated ratings, the highly-rated trials and the low-rated trials contain identical sets of stimuli (each contributed by a different observer to each set). This was therefore our motivation in creating a stimulus set that generated highly individualized responses: rating-specific neural responses would then not be attributable to the features of any particular artworks, thus allowing us to isolate neural correlates of the aesthetic experience itself.

**Figure 2:**
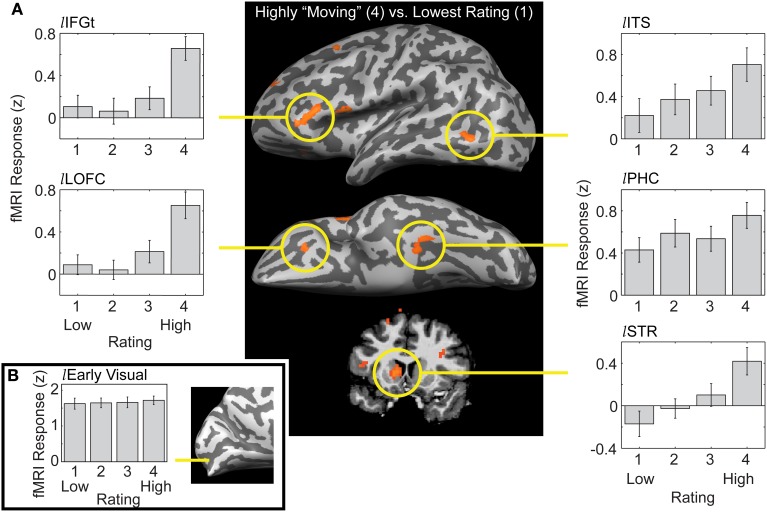
**Distinct patterns of response to artworks as a function of their ratings in a distributed network of brain regions. (A)** *Center panel*: a whole-brain analysis contrasting trials on which observers rated artworks as highly moving (4) vs. trials where artworks were given the lowest rating (1), showing a lateral (top) and ventral (middle) view of an inflated left hemisphere, and a coronal section (bottom) through the striatum (data thresholded at a False Discovery Rate of *q* < 0.05 in volumetric space and projected on a hemisphere of a single observer for visualization). *Right-side panels*: a linear increase with rating was observed for the activation loci in occipitotemporal cortex and some subcortical loci (shown here: left inferior temporal sulcus, *l*ITS; left parahippocampal cortex, *l*PHC; left striatum, *l*STR; see (Vessel et al., 2012) for additional ROIs and further detail). *Left-side panels*: a nonlinear, “step”-like response pattern was observed in the anterior activation loci; responses did not differ for images rated 1, 2, or 3, but were significantly elevated for images rated 4 (shown here: left inferior frontal gyrus *par triangularis*, *l*IFGt; left lateral orbitofrontal cortex, *l*LOFC). **(B)** Extensive portions of early visual cortex were strongly activated by all paintings, but the magnitude of fMRI response did not differ by rating.

We performed several different analyses, using both statistical activation maps and regions of interest (ROIs) generated from the same data set or from separate “localizer” runs. We first created whole-brain activation maps by contrasting the group-level brain response to the most moving trials (rated as “4”) with the responses to the least moving trials (rated as “1”). This “4-vs.-1” analysis revealed a network of regions distributed across posterior, anterior, and subcortical structures (Figure 2A; note that, in addition, extensive portions of visual sensory cortex were strongly activated by all stimuli, but the magnitude of response did not differ by rating; Figure 2B). This is consistent with conclusions from previous research using a variety of stimuli that multiple brain regions are engaged during aesthetic appreciation (Aharon et al., 2001; Blood and Zatorre, 2001; Cela-Conde et al., 2004; Kawabata and Zeki, 2004; Vartanian and Goel, 2004; Jacobsen et al., 2006; Koelsch et al., 2006; Di Dio et al., 2007; Kim et al., 2007; Yue et al., 2007; Calvo-Merino et al., 2008; Fairhall and Ishai, 2008; Cupchik et al., 2009; Ishizu and Zeki, 2011; Lacey et al., 2011; Salimpoor et al., 2011; Jacobs et al., 2012; Kuhn and Gallinat, 2012). Note that the large inter-observer variability in behavioral responses to our stimulus set means that the common (group-level) activation in the 4-vs.-1 contrast must reflect effects of the aesthetic experience itself, i.e., it could not be due to any attributes of particular art stimuli that gave rise to this experience. This is because, at the group level, the set of highly rated trials consisted mostly of the same images as the poorly rated trials (recall that for every image rated as high by one observer there was, on average, another observer that rated it as low). This also means, however, that our approach is more restrictive than that in some other studies, which could give rise to differences in the activations observed. We will not go here into details of comparing and contrasting the loci of activation with those previously reported in the literature (see Vessel et al., 2012). Instead, we focus below on those aspects most relevant for a novel and intriguing finding: the activation by highly moving stimuli of the default mode network (DMN).

The bar graphs surrounding the activation map in Figure 2A show fMRI response magnitude as a function of observers' ratings for select ROIs, revealing that different ROIs exhibited distinct response patterns. Moreover, ROIs could be grouped in two main categories: for one set of ROIs, response magnitudes varied linearly with rating (right-side panels: *l*ITS, *l*PHC, and *l*STR). The linear response pattern was observed in different variations in terms of its relation to the baseline (“rest”) level: in occipitotemporal cortex, higher ratings were accompanied by linearly changing BOLD signals that either increased well above a resting baseline (*l*ITS, and *l*PHC) or, in one case, decreased well below it (*r*STG, not shown). In subcortical regions, fMRI activity was suppressed below its resting level for low-rated stimuli and rose progressively to above-rest for highly rated stimuli [*l*STR, bottom right panel; PRF, not shown; see Vessel et al. (2012) for ROIs not shown here and further details]. Since the 4-vs.-1 contrast selects for regions that responded differently to trials rated “4” compared with trials rated “1,” the pattern of response for the intermediate ratings of 2 or 3 in these regions is *a priori* unknown. It is therefore noteworthy that responses in these ROIs followed a linear trend so closely. Moreover, regions whose response patterns were significantly *non-linear* all showed the same distinct pattern, as follows.

**Figure 3:**
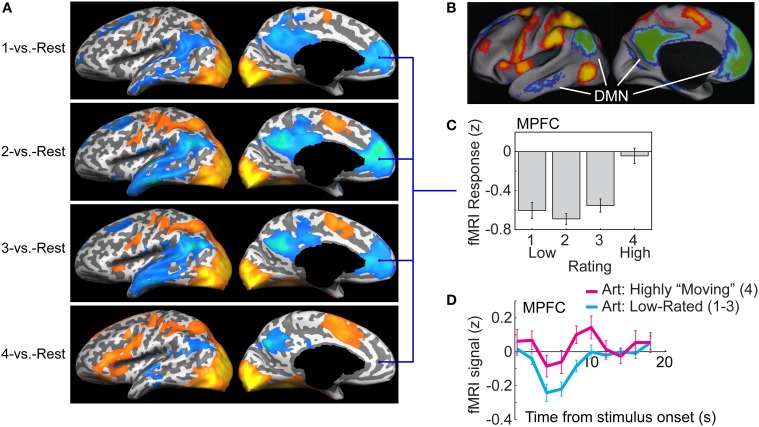
**The default mode network (DMN) deactivation during task performance is alleviated when viewing highly moving artworks. (A)** Lateral (left) and medial (right) views of an inflated cortical surface are overlaid with statistical maps comparing fMRI responses during task (viewing and rating of artworks) vs. “rest” periods. Maps were computed separately for trials from each of the four possible ratings, 1 (top) to 4 (bottom). The warm colors indicate greater fMRI response during task; the cool colors indicate greater response during rest (“deactivation”; data were thresholded at a False Discovery Rate of *q* < 0.05 before projection onto one observer's inflated cortex). In trials rated 1, 2, or 3 (top three panels) there were deactivations in medial prefrontal cortex (MPFC), posterior cingulate cortex (PCC), lateral temporal cortex (LTC), temporoparietal junction (TPJ), and superior frontal gyrus (SFG). The suppression was greatly reduced for the highest-rated trials (4; bottom panel). **(B)** The spatial pattern of deactivation during the lower-rated trials (1–3) closely resembles that of the default mode network [DMN; image adapted with permission from Fox et al. (2005) Copyright 2005 National Academy of Sciences, U.S.A.]. **(C)** Average fMRI response in the MPFC region of interest (ROI) was markedly and uniformly below rest for trials rated 1, 2, or 3, but was not different from rest for the highest-rated trials (4). **(D)** fMRI signal timecourse in the MPFC for the lower-rated trials (cyan) and the highest-rated trials (magenta). Note that activity initially fell below its level during rest also for the highest rated trials, yet it rapidly recovered and then proceeded to increase above rest level. The fMRI response used for both C and D was estimated from an ROI defined via a contrast of the response on “4” trials vs. the other trials (4-vs.-321), conjoined with a map of regions showing no difference in the low-rated trials. The timecourses for each rating level were extracted by modeling the average timecourse from this ROI as a set of four finite impulse response functions (Dale and Buckner, 1997).

A second category of regions revealed by the 4-vs.-1 contrast were characterized by a distinct “step” pattern: fMRI responses in those regions did not differ significantly for images rated 1, 2, or 3; only for the highest (4) rating was there a significant difference in response magnitude, and it was marked and dramatic (Figure 2A, left-side panels; see Vessel et al., 2012 for other examples; see also below, Figure 3). We performed several additional analyses in order to examine more closely the nature and spatial distribution of these nonlinear “step” responses. A whole-brain analysis contrasting the highest-rated trials with an average of all other trials (4-vs.-321; Vessel et al., 2012) gave us more power to detect regions that may not have reached the significance threshold in the 4-vs.-1 contrast due to the lower number of trials. A conjunction was subsequently computed to specifically capture the regions that, while showing a differential response to the highest-rated stimuli (“4”), showed no significant differences in responses within the lower ratings (1, 2, and 3). The resulting statistical map contained large swaths of highly significant differences in several regions known to be part of the DMN, and further examination indicated that the pattern of responses in those regions consisted of a strong *de*activation in trials rated 1, 2, or 3 (with no significant differences in magnitude), which was greatly alleviated or even eliminated in the highest-rated trials [“4”; see Vessel et al. (2012), Figure 6]. To better underscore the commonalities and differences from what is currently known about the DMN, below we represent our results in a different format than before, which is modeled after that used in the DMN literature.

Figure 3A shows statistical activation maps contrasting the task-induced fMRI responses with “Rest”—intervals interspersed between the trials when only a blank screen was shown—overlaid on the inflated surface of the left hemisphere. The maps were generated separately for each of the four sets of trials corresponding to the four possible ratings (from top to bottom: 1-vs.-Rest to 4-vs.-Rest). Large regions in occipital cortex, as well as portions of parietal and frontal cortex, showed activation above rest for all four rating levels (warm colors, red-yellow). The cool colors (blue–green) denote regions that showed a *reduced* fMRI signal during viewing and rating of the artworks, compared to during rest. For the sets of trials rated 1, 2, or 3 (top three panels), extensive regions of reduced activity can be seen; their anatomical loci and spatial distribution closely resembles that observed in studies that contrasted activity during a wide range of cognitive and perceptual tasks with periods of rest (Shulman et al., 1997; Simpson et al., 2001), shown in Figure 3B (adapted from Fox et al., 2005). Specifically, reduced activity was observed in the medial prefrontal cortex (MPFC), posterior cingulate cortex (PCC), precuneus (PCu), temporo-parietal junction (TPJ), lateral temporal cortex (LTC) and superior frontal gyrus (SFG). Studies of blood flow and oxygen utilization indicate that the baseline level of these regions—that measured during rest—corresponds not to a lack of activity, but rather to activity associated with an ongoing, organized “default mode” of brain processing, which is suspended during performance of tasks that require externally directed attention (Gusnard and Raichle, 2001; Raichle et al., 2001). The reduced fMRI response in regions of this **default mode network** (DMN) during task performance is therefore widely referred to as *deactivation* (although the mechanisms giving rise to it are not fully understood).

KEY CONCEPT 3 | Default mode network A network of brain regions typically found to be suppressed when observers engage in externally oriented tasks, which includes the medial prefrontal cortex (MPFC), posterior cingulate cortex (PCC), temporo-parietal junction (TPJ), lateral temporal cortex (LTC), superior frontal gyrus (SFG) and the hippocampus. Patterns of spatial correlation measured in the absence of directed tasks (resting state fMRI) support this network structure and suggest that the DMN is composed of midline hub regions (MPFC, PCC) and two subsystems.

In contrast with the pattern observed for trials rated 1, 2, and 3, DMN regions showed markedly less deactivation during the highest-rated trials (“4”; bottom panel in Figure 3A). Indeed, in some portions of the DMN—most notably, in the MPFC—the deactivation seems all but gone. ROI analysis confirmed that the MPFC was strongly and uniformly deactivated for lower-rated trials (1–3), but not at all during those trials when the artworks were given the highest rating (4), resulting in a step-like response pattern [Figure 3C; for plots of several other DMN components, see Vessel et al. (2012)].

## The default mode network and self-referential mental processing

A defining characteristic of the DMN—indeed, how it was discovered—is that it is suppressed when observers are engaged in demanding tasks that require them to focus on external stimuli, compared with its level of activity during passive viewing or periods of rest between the tasks (Shulman et al., 1997; Buckner et al., 2008). The ubiquity of DMN deactivation during many different cognitive tasks with a variety of stimuli and response demands, along with studies of functional connectivity during rest, have led to the view that the DMN represents a “task-negative” network of brain regions that normally functions in an anti-correlated manner from “task-positive” networks such as sensory-semantic pathways and the dorsal attention network (Ingvar, 1979; Corbetta and Shulman, 2002; Fox et al., 2005; Buckner and Carroll, 2007). The finding that, in our own task, the cortical regions that overlap with previously identified components of the DMN (MPFC, PCC, TPJ, LTC) showed significant deactivation below their baseline (rest) level during a majority of the trials, those rated 1–3 (Figure 3A, top three panels) is therefore consistent with what is known about the DMN. From this same perspective, the dramatic reduction of deactivation in the trials rated “4” (Figure 3A, bottom panel) and its complete absence in the MPFC (Figure 3C) therefore seems puzzling. But consideration of additional findings about the DMN offers a potential explanation.

Following its initial identification, further research showed that the DMN regions can maintain their baseline activity not only during periods of (waking) rest, but that they can escape deactivation, or even become activated above baseline, also during the performance of structured tasks. Ventral portions of the MPFC are involved in affective decision making processes, including (but not restricted to) encoding the subjective value of future rewards and assessing the emotional salience of stimuli (Bechara et al., 1999; Knutson et al., 2005; Kringelbach, 2005; Kable and Glimcher, 2007; Schmitz and Johnson, 2007; Levy and Glimcher, 2011). The anterior and dorsal portions of MPFC are active in tasks involving self-knowledge such as making judgments about oneself as well as about close others (family and friends), self-relevant moral decision-making (Reniers et al., 2012) and in “theory of mind” tasks that require gauging others' perspectives (Zysset et al., 2002; Ochsner et al., 2004, 2005; Amodio and Frith, 2006; Mitchell et al., 2006; Enzi et al., 2009; Andrews-Hanna et al., 2010; Whitfield-Gabrieli et al., 2011). The PCC and medial temporal lobe regions are active during tasks that involve retrieving autobiographical memories as well as planning or simulating the future (Buckner and Carroll, 2007; Buckner et al., 2008; Andrews-Hanna et al., 2010).

The DMN is thus emerging as a highly interconnected network of brain regions that support **self-referential mental processing** (Northoff et al., 2006). Such processing is, of course, ubiquitous in everyday life and is undoubtedly important for normal functioning. In experimental settings it can occur spontaneously (e.g., as “mind wandering” during periods of rest) but it can also be triggered in structured tasks, by external stimuli that cause observers to draw on self-referential information (intentionally or automatically), or to engage in inwardly focused attention. Could this have been the case with the images that our observers rated as “highly moving”? We propose that the answer is yes, as detailed in the account provided below.

KEY CONCEPT 4 | DMN and self-referential mental processing Structured tasks can activate the DMN if they require some self-referential processing (e.g., introspection, autobiographical memory recall). Similarly, it is presumed that the DMN is metabolically active during baseline non-task periods (e.g., fixation or “rest” conditions) because observers engage in such processes spontaneously.

## Intense aesthetic experience: a (non-personal) external stimulus reaches the self

Taste in art is highly individual and can be hard to predict by even the most well-informed bystander (e.g., Bell and Koren, 2007), yet it is strongly felt. Indeed, many individuals consider their artistic taste to be an important part of their identity, their sense of *who they are*. This is not limited to connoisseurs of “high art”: from teenagers whose tumultuous struggles for self-determination are conducted to the soundtrack of meticulously compiled music collections, to adults of all ages who repeatedly turn to their favorite genres of fiction or film to escape the tedium of their daily lives, our taste in art is intertwined with the choices we make about how to spend our time and with whom to spend it, and as such it is part of who we are. How does this come about? What gives certain artworks their mysterious “pull”? Our data say nothing about this in terms of the attributes of the artwork itself. (Whether this will remain a mystery forever or may yield to future research is an interesting question that will not be discussed here). But our results suggest that the strong effect of certain artworks *can* be understood in terms of the physiological state they generate and how this state is experienced, or interpreted, by the observer.

We propose that certain artworks can “resonate” with an individual's sense of self in a manner that has well-defined physiological correlates and consequences: the neural representations of those external stimuli obtain access to the neural substrates and processes concerned with the self—namely to regions of the DMN. This access, which other external stimuli normally do not obtain, allows the representation of the artwork to interact with the neural processes related to the self, affect them, and possibly even be incorporated into them (i.e., into the future, evolving representation of self). This hypothesis gains considerable support from the way that the fMRI responses evolved over time in the MPFC, the region most associated with evaluations of self-relevance. As can be seen from the time course plots in Figure 3D, immediately following stimulus presentation the fMRI signal in the MPFC fell below baseline for all images, i.e., also for those images that were (later) rated by the observer as highly moving (4). Thus, the initial predisposition of this DMN region was, for *all* external stimuli, to deactivate. But in contrast with the MPFC response to the artworks rated 1, 2, or 3, which was suppressed during image presentation and remained below baseline throughout the subsequent recovery (Figure 3D, cyan line), in the 4-rated trials activity started recovering soon after stimulus presentation and then continued to rise *above* baseline (magenta line). This is reminiscent of the MPFC recovery from deactivation observed when a highly self-relevant stimulus such as one's own name is presented in a stream of self-irrelevant stimulation, as in the “cocktail party effect” (e.g., Cherry, 1953; Bargh, 1982; Wood and Cowan, 1995; Perrin et al., 2005). But why should a hitherto unseen artwork, that has no *a priori* personal relevance for the observer, have this effect of engaging the DMN system? Again, we cannot say what attributes make specific artworks so exquisitely attuned to an individual's unique makeup. And yet this hypothesis provides a coherent explanation of our data in that it is consistent not only with what we know about the DMN, but also with what we know about art.

Great art is, almost by definition, universal: the wide appeal it commands comes from a connection with fundamental aspects of human nature and human cognition (Kant, 1790/1987). Yet, at its best, art in any of its forms—visual art, music, literature, etc.—can feel strikingly personal. Intense aesthetic experience often carries with it a sense of intimacy, “belonging,” and closeness with the artwork. It may be hard to imagine that the experiences of our observers, lying in an MRI scanner watching images of little-known artworks selected by an experimenter who knew nothing about them, reached the profound levels that give art its intense power. And yet the data are compellingly in line with the phenomenology of aesthetic experience: in the small subset of the trials that observers rated as “highly moving,” DMN regions and in particular the MPFC were released from deactivation and even activated above baseline, a hallmark of self-relevant neural processing. Perhaps the key to this was in our experimental design, which relied on a stimulus set that maximized individual differences in behavioral response. As already mentioned, the original motivation for this design was to measure neural correlates of aesthetic experience in the absence of potential confounds with effects of stimulus attributes. But the emphasis on a diversity of artistic styles and topics may have, serendipitously, also increased the chances that a few of the artworks resonate with each observer in a particularly powerful way.

Note that the “resonance” between certain artworks and observers' sense of self that, we propose, occurs during intense aesthetic experience, is different from explicitly self-referential emotions such as pride, shame, guilt and embarrassment, as these involve an appraisal of self-responsibility for an event (Silvia, 2012). It is also interesting to note in this context that intense aesthetic experience can sometimes be thrillingly bidirectional: not only does the perceiver feel as if they understand the artwork, but there is a sense that the artwork “understands” the perceiver, expressing one's own innermost thoughts, feelings, or values. The latter sense points to the possibility that it is the artist, not the artwork, who has understood something deep about the perceiver's experience; hence the intensely personal connection felt by many people toward favorite artists who are, after all, strangers to them. In some cases, this bidirectionality is accompanied by a perceived or real congruence with the intentions of the artist (Jucker and Barrett, 2011; Tinio, 2013). Thus, unlike in self-referential emotions, in aesthetic experience the relation to others is not focused on appraisal but on a sense of understanding, gained insight and meaning. The extraction of meaning has been suggested previously as a primary factor of aesthetic experience (Martindale, 1984; Leder et al., 2004). But, while those authors suggest that an appeal to self-related information is but one way in which viewers extract meaning from artwork, the release of the DMN from suppression on only the trials rated “4” suggests that, in fact, self-relevance is an integral aspect of intensely moving aesthetic experience.

What internal signal did the observers use to provide their responses? It is tempting to think that they were able to detect the unusual release from deactivation in the DMN when viewing artworks which they (later) rated “highly moving,” and that they based their responses on this internal signal. Indeed, the MPFC and PCC respond to self-relevant information even when there is no explicit requirement to evaluate self-relevance, and such information is in fact task-irrelevant (Moran et al., 2009; Reniers et al., 2012). Perhaps observers conferred the highest rating on those artworks that invoked in them a sense of self-relevance, even though they were not instructed to do so, and may well be unable to explicitly state this as their strategy. Yet given the poor temporal information provided by fMRI, it is too early to rule out the possibility that responses on the “4” trials arose from posterior regions whose activity grew linearly with rating or from other frontal regions that showed positive activation for only the “4” trials, and that the release from suppression in the DMN for highly moving artworks occurred subsequent to the evaluation. A recent MEG study of aesthetic appreciation reported coherence between frontal midline, posterior and temporal regions that was detectable 1 s after onset of images deemed “beautiful” (1000–1500 ms analysis window) but not in an earlier epoch (250–750 ms; Cela-Conde et al., 2013). This finding is consistent with our proposal that the release of the DMN from suppression for intensely moving artworks occurs subsequent to an initial perceptual and semantic analysis, and early enough to be a potential basis for response selection; however, it leaves open the question of how, in time, explicit evaluation relates to these dynamics.

A coactivation of the DMN and stimulus-driven sensory system as we have observed for strongly moving aesthetic experiences has so far not been reported in other contexts. Yet, if our self identity is to be influenced by the world we inhabit, it may be that similar moments should occur with greater frequency than would be expected based on the current conceptualization of the DMN as a network that is invariably suppressed during mental activity which is directed at the external world. It may be that our findings are just the “tip of the iceberg”—i.e., that instances of resonance between external stimuli and internal, self-related processing are more commonplace in daily life than what has so far been captured in fMRI experiments in the laboratory. By that view, much of our existence may be well-served by switching between periods of dominance of externally-directed (“task-positive”) brain networks over the DMN and vice versa, but those periods are punctuated by significant moments when our brains detect a certain “harmony” between the external world and our internal representation of the self—allowing the two systems to co-activate, interact, influence and reshape each other.

### Conflict of interest statement

The authors declare that the research was conducted in the absence of any commercial or financial relationships that could be construed as a potential conflict of interest.
